# Temporal dynamics of ammonia and nitrous oxide emissions following green crop residue recycling in soils

**DOI:** 10.1038/s41598-026-54279-5

**Published:** 2026-06-12

**Authors:** Varunesh Chandra, Gwenaëlle Lashermes, Patricia Laville, Alain Fortineau, Benjamin Loubet, Raia Silvia Massad

**Affiliations:** 1 Université Paris-Saclay, INRAE, AgroParisTech, UMR EcoSys, 91120 Palaiseau, France; 2 Université de Reims Champagne-Ardenne, INRAE, FARE, UMR A614, Reims, France

**Keywords:** Soil organic matter, Laboratory incubations, Soil pH, Red clover, Mineral nitrogen dynamics, Greenhouse gas mitigation, Biogeochemistry, Climate sciences, Ecology, Ecology, Environmental sciences

## Abstract

The recycling of green crop residues is promoted for enhancing soil organic carbon sequestration and health, but can also stimulate emissions of nitrous oxide (N₂O) and ammonia (NH₃). Soil tillage can lead to different residue distributions which affect contact with decomposers and local gas and solute diffusion. This study aims to quantify N₂O, NH₃, and carbon dioxide emissions from N-rich red clover residue subjected to three different distributions in the soil—surface, layered, and mixed—across three contrasting agricultural soils. The incubations were performed under laboratory conditions for 50 days at 15 °C, with a water-filled pore space of 60%. Gas fluxes as well as soil ammonium and nitrate contents were measured. Results showed that N₂O emissions were strongly influenced by soil type and residue placement, with sandy loam producing the highest cumulative fluxes, particularly in the mixed (17.4 kg N ha⁻^1^) and layered (11.5 kg N ha⁻^1^) treatments. NH₃ volatilization occurred almost exclusively from surface residues, peaking at 10.2 kg N ha⁻^1^ in sandy loam soil. Emission for N_2_O were significantly higher than previously reported for residues. These findings highlight that both residue placement and soil properties critically determine gaseous emission patterns.

## Introduction

The recycling of crop residues into soil is increasingly recognized as a crucial management practice for reducing synthetic fertilizer use, enhancing soil organic carbon (SOC) content, and improving overall soil health^1,2^. Cover cropping and the introduction of ley grass into rotations has significant potential to increase SOC in soils, thus mitigating agricultural greenhouse gas (GHG) emissions^3^. Despite these benefits, the recycling of organic materials such as crop residues also introduces nitrogen (N) into the soil, potentially increasing emissions of ammonia (NH_3_) and nitrous oxide (N_2_O)^4–8^. This is particularly true for residues from green cover crops and ley grass but also N-rich plants such as legumes^9,10^. Ammonia is a significant atmospheric pollutant and a precursor of atmospheric particulate matter (PM10 and PM2.5) (ex^11,12^). Nitrous oxide, on the other hand, is a potent long-lived GHG with a high global warming potential and contributes to stratospheric ozone depletion.

Organic residue decomposition not only supplies mineral nitrogen but also stimulates microbial respiration, leading to the release of CO_2_ from soils. The extent of this respiratory loss depends on residue composition, soil texture, and the degree of residue incorporation, with recent reviews and meta-analyses confirming that residue management can markedly modify greenhouse gas emissions at the soil scale^13,14^. In this context, CO_2_ fluxes are an important companion variable to NH_3_ and N_2_O, as they help characterize the decomposition “hot moments” associated with residue recycling.

The decomposition of organic matter releases mineral N, primarily in the form of NH_4_^+^. Ammonia volatilization occurs when NH_4_^+^ ions are converted to NH_3_ gas, a physicochemical process governed by acid–base and thermodynamic equilibria influenced by soil pH and temperature. Concurrently, NH_4_^+^ ions fuel various microbial pathways that convert NH_4_^+^ to nitrate (NO_3_^-^) and ultimately to N_2_O through processes such as nitrification–denitrification, co-denitrification, or nitrifier denitrification^15^. These complex microbial processes are highly influenced by a range of environmental and soil factors, including C and N availability, temperature, soil moisture, pH, and inherent soil physical and chemical properties^16–19^. Numerous studies have reported the effects of climate (precipitation and temperature), soil aeration, and soil type on field-level N_2_O emissions and NH_3_ volatilization from soils following manure, slurry, or fertilizer application^20^^21,22^. Soil tilling, which primarily redistributes crop residues within the soil profile, has a strong influence on local conditions for gas and solute exchange, as well as on the contact between soil and microbial decomposers^23^^,24^. In conservation agriculture, where no tillage is performed, residues remain on the soil surface as mulch^25^. Under reduced tillage, residues are partially incorporated into the soil, whereas with full inversion tillage, they are buried and form a distinct layer within the soil profile^26^.

While the drivers of these emissions are well-established, gaining a better understanding of the temporal dynamics of N_2_O and NH_3_ emissions in relation to the decomposition process is essential for identifying critical “hot moments” in agricultural fields^27,28^. N_2_O emission peaks primarily occur within a month of N fertilizer application, though the onset is not always immediate, and significant emissions often materialize only after rainfall, which triggers or reactivates underlying microbial processes. The amplitude and duration of an emission peak are highly variable depending on pedo-climatic conditions and fertilizer types. Existing data on NH_3_ volatilization from surface-applied crop residues have been reported for various arable crops^29^ grass^30,31^, and green manure crops/mulches^32^. The decomposition of crop residue left on the field can emit NH_3_^33^, with estimates suggesting a substantial contribution to national NH_3_ budgets^29^. Some studies report that incorporating residues into the soil significantly reduces, or can even virtually eliminate, NH_3_ volatilization compared to surface placement^34,35^. However, direct contact between plant material and soil can initially increase NH_3_ volatilization due to more rapid microbial proliferation, though this difference tends to diminish over time^26^. Measurements by Francis et al.^36^ for example report NH_3_ volatilization after cauliflower incorporation. Recent laboratory measurements by Janz et al.^4^ report an increase of 25.1% of NH_3_ emissions after incorporation.

More importantly, the quantification of these emissions from residues is associated with high uncertainties related to the quantity of residues left on the soil after harvesting as well as the variability linked to different crop species, soils, climate, and management practices^37^. Extensive research has examined the impact of different crop residue types on gaseous emissions (e.g^38–40^). Labile crop residues, such as green residues from cover crops or ley grass, decompose very rapidly in the soil, releasing significantly important amounts of mineral N^9,10,41,42^. The spatial distribution of crop residues in soil is another key factor affecting N_2_O emissions^29,43^^,44^. As organic matter decomposition rates directly affect soil N mineralization, the positioning of crop residues within the soil is a critical determinant of N_2_O emissions^45–48^. Residues incorporated into the soil create “hot spots” for microbial processes like nitrification and denitrification and are more prone to decomposition due to enhanced microbial contact compared to surface residues^49^,^26,50,51^.

This study aims to quantify the dynamics of N_2_O and NH_3_ emissions from a N-rich and green residue (specifically red clover) decomposing into 3 agricultural soils under different vertical distribution patterns in a controlled laboratory setting over a 50-day period. In addition to gas emissions, soil mineral N and C contents, along with CO_2_ fluxes, were measured throughout the incubation process. Three different residue placement methods—mixed (maximum soil contact), layered (intermediate contact), and surface (minimal contact)—were tested across three soil types to provide a specific examination of the interaction between residue placement and soil characteristics.

Our initial hypothesis is that the temporal dynamics and magnitude of N_2_O and NH_3_ emissions from N-rich green crop residues (red clover) will vary significantly with different vertical distribution patterns (mixed, layered, surface) and across different soil types in a controlled laboratory setting, with surface-applied residues generating higher NH₃ emissions, while incorporated residues having higher N₂O emissions.

## Materials and methods

### Soil and residues

Soils were sampled in spring 2018 from 0–20 cm depth from three different locations to prepare soil microcosms for laboratory incubation. The first soil is a slightly acidic sandy loam and was collected from an experimental site in Alnarp, Sweden (55°39′28.2"N 13°64′56.1"E) (called SLU here). Two soils, one with high calcareous content and therefore high pH and the other with neutral pH were sampled from Thiverval-Grignon, France (48°50′53.4"N, 1°56′23.1"E and 48°50′37.0"N 1°57′03.6"E respectively). These soils are called GEXER and GICOS respectively. The GICOS soil was sampled in the ICOS FR-Gri site (https://meta.icos-cp.eu/resources/stations/ES_FR-Gri).

All soils were sieved to 6 mm and all the vegetation fragments were removed. They were sampled for analysis of total C and N, pH and texture prior to its storage. Table 1 shows the physico-chemical properties as well as the cropping history of these soils.

**Table 1:** Physico-chemical characteristics and cropping history of the three soils – SLU, GEXER and GICOS.

*Soil*	***Units***	***SLU***	***GEXER***	***GICOS***
*Clay (*< *2 µm)*	g kg^−1^	158	214	108
*Fine silt (2/20 µm)*	g kg^−1^	122	139	287
*Coarse silt (20/50 µm)*	g kg^−1^	102	219	353
*Fine sand (50/200 µm)*	g kg^−1^	307	100	104
*Coarse sand (200/2000 µm)*	g kg^−1^	311	47	27
*Organic C*	g kg^−1^	15	18.4	20.8
*Total N*	g kg^−1^	1.49	1.79	2.11
*CaCO*_*3*_	g kg^−1^	< 1	275	58
*Organic matter*	g kg^−1^	26	31.9	41.7
*C:N*	-	10.1	10.3	9.9
*Water pH*	-	6.2	8.3	7.6
*CEC*	cmol kg^−1^	15.5	13.6	15.5
*Cropping history*		Bare soil with beetroot previous year and no cover crop	Barley with winter wheat in the previous year	Winter wheat with rapeseed in the previous year

Red clover plants (C:N = 17.9, Dry matter 2.5%, N-NO_3_ ‰ = 0, N-NH_4_ ‰ = 0.2, total N = 2.5 ± 0.0% DM, total C = 41.9 ± 0.3% DM) sampled from Ås, Norway (59°39′57.7"N, 0°46′06.5"E) were harvested at a reproductive stage. Different organs were separated (leaves, stems, flowers and petioles), weighed and dried for a week at 40 °C and then cut to 1 cm pieces. These parts were then mixed together in proportions similar to proportions of field occurring plants (7% stem, 34% flowers and leaves, 29% petioles) for the incubation.

### Soil microcosms

All soils were preincubated for one week at 15 °C in a temperature regulated chamber with aeration. SLU soil was preincubated at 35% water filled pore space (WFPS), GEXER and GICOS at 40%. The difference between the soils is due to soil initial water content. We complemented each soil with potassium nitrate (KNO_3_ 98% pure) before the preincubation stage to reach 100 mg NO_3_^-^ N kg^−1^ soil corresponding to approximately 100 kg N ha^−1^. This concentration was chosen as an experimental approach to avoid nitrogen limitation and to allow faster mineralization of organic material. Soils were then packed at 1.25 g cm^−3^ bulk density in incubation cylinders of 12.5 cm diameter with perforated bottom. This bulk density was used as a standardized packing density across all soils to ensure comparable microcosm structure and water-filled pore space conditions. We did not intend this value to represent the exact field bulk density of each site. To ensure homogeneity in the cylinders and the different modalities we were planning, soils were put in layers of 2 cm each until they reached 8 cm height in the cylinder. Mass of each 2 cm layer was calculated from bulk density, cylinder volume and moisture content and compacted to 2 cm to obtain a WFPS of 60% before it was topped with another 2 cm layer. Prepared 1 cm cut residues were added with an amount of 0.04 g DM cm^−2^ surface of soil to reach an equivalent in nitrogen of 100 kg N ha^−1^, the added total C to the soil is 1676 kg C ha ^−1^. The residues were either on top of the 8 cm cylinder (surface modality), or after having added the first two layers (at 4 cm) for the sandwich modality or by mixing them with the upper two layers of soils for the mixed modality. No residue was added to control soil; they were amended with the equivalent of 100 kg N ha^−1^ of KNO_3_. Three repetitions of each modality and control were prepared. Similar microcosms with cylinders of perforated bases (7 cm diameter) were prepared at the same time with preincubated soils and residues in accordance with Table 2 for mineral N extraction and sampling. All incubations were performed at 15 °C for 50 days. Each prepared microcosm was covered with a perforated film and kept in an aerated dark room when not in measurement. One point to note here is that there was no surface modality for GEXER soil due to lack of appropriate soil availability.

**Table 2:** Modalities with different vertical distribution of residues in soils.

Modalities				
Soil type	GEXER SLU GICOS	GEXER SLU GICOS	SLU GICOS	GEXER SLU GICOS
Added total N	100 kg N /ha	100 kg N /ha	100 kg N /ha	100 kg N /ha
Added total C	1676 kg C/ha	1676 kg C/ha	1676 kg C/ha	-
WFPS	60%	60%	60%	60%

### Measuring gas emissions from soil microcosms

We set up a gas incubator (Gas Emission Detection Incubator: GEDI) allowing us to measure gas fluxes from soil cylinders. The system allows the measurement of four different gases: N_2_O, NH_3_, CO_2_ and H_2_O vapor.

**Fig. 1:**
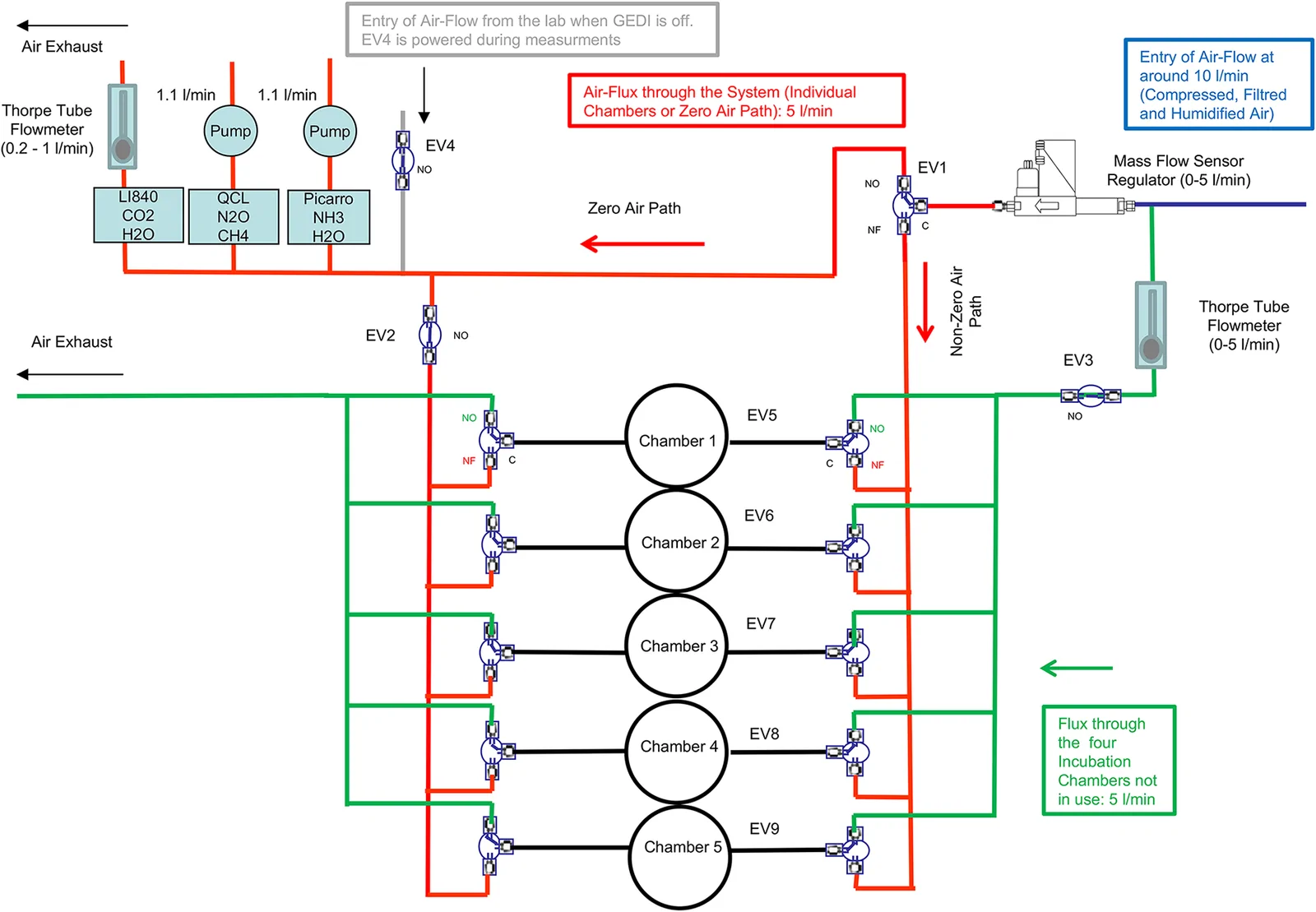
Schematic showing flow circuitry of Gas Emission Detection Incubator. Red circuit represents the main measurement airflow circulation. Green circuit represents the auxiliary “sweeping” airflow circulation. EV stands for electro valves, NO for normally open and NF for normally closed.

GEDI (see Fig. 1 for more details) is a system that allows for automatic measurements of gas exchanges in a laboratory setup. It is all setup in a temperature-controlled box (Length: 115 cm, Width: 60 cm, Height: 60 cm) with five chambers with each chamber holding one soil cylinder. The chambers are exclusively made of glass and all tubing was Teflon PTFE made to minimize NH_3_ adsorption. Chambers were also cylindrical with a headspace of 530 cm^3^. A Teflon based air deflector was used^52^ for the chambers to (i) allow turbulent air flow in the head space and (ii) avoid direct air flow on the soil so that it does not dry.

All of the chambers are connected in parallel to one another through three-way solenoid valves at each inlet and outlet. Air is supplied to each of these chambers in sequence from their inlets to allow for a gas flow through the outlet. Gas flow from the outlet then reaches the different analyzers for measurement. The three-way solenoid valves are also connected to another parallel air circuit simultaneously that allows for constant airflow in the chambers not in measurement through a secondary exhaust preventing any accumulation inside those chambers. In Fig. 1, the red circuit corresponds to the measurement circuit, and the green to the auxiliary circuit. The gas sampling is performed automatically following a sampling sequence controlled by a data logger (Campbell CR1000X). Air flow through the system is controlled and measured by a mass flowmeter. Fluxes are computed following a steady-state chamber approach, i.e. from the difference in air trace-gas concentrations between the outlets of a cell with treated soil-cylinder and a blank-cell. The soil-headspace exchange rate of each trace-gas is a result of the mass balance between the two outlets (chamber air) concentrations assuming mass flow equilibrium conditions.

1 $${F_{ss}} = {\rm{ }}Q/A \times {\rm{ }}\left( {{C_{out}} - {\rm{ }}{C_{blk}}} \right)$$

where *F*_*ss*_ stands for the trace gas flux; *A* denotes the soil surface of the soil cylinder; *Q* is the headspace air flow rate. *C*_*blk*_ and *C*_*out*_ are the trace gas mixing ratios of the outflowing chamber air for the blank cell and cell with treatment respectively. Fluxes were converted to kg N (or C)/ha/d using air temperature measurements (the atmospheric pressure was set to 1013 hPa).

The air supplied for measurement is ‘cleaned’ by filter traps connected in series at the inlet of the GEDI. Manganese dioxide-based filter (Purafil Inc., Atlanta, GA, USA) to eliminate ozone and nitrogen oxides (NO_x_), active charcoal filter to trap volatile organic compounds (VOCs), acetic acid based filter to capture background NH_3_, a cotton-tissue based particle filter and an empty trap to obstruct entry of water to the incubator. A bubbling humidifier with ultra-pure water is connected in series to these filters to maintain constant air humidity in the incubator. All the measurements took place in the dark. An airflow of 4.0 l min^−1^ was maintained at the entry of GEDI. Temperature was regulated with a Peltier system with a constant value of 15 °C. A datalogger (Campbell CR1000X) was used to record measured data. Thermocouples were attached to this datalogger to record real time temperature of the incubator.

A measurement cycle for the five cylinders placed in the GEDI is 140 min. Cycle starts with a 15 min purge of the whole system with clean air, followed by a sequential measurement of each cylinder for 25 min.

Three gas analyzers were connected in parallel with the GEDI outlet. A constant air-flow rate (1 L min^−1^) was maintained at the input of each analyzer by a Thorpe tube flow-meter connected in series with GEDI [See Fig. 1 for more details]. An NH_3_ Cavity Ring-down Spectroscopy Analyzer (PICARRO G2103) was used for measurement of NH_3_ (in ppb) and water (in % by mass). Infrared analyzer LICOR (Li – 840 A) was deployed for continuous monitoring of CO_2_ (in ppm). It also detected water concentration in parts per thousand (ppth). A Quantum Cascade Laser Tunable Infrared Laser Differential Absorption Spectrometer (QCL TILDAS – Aerodyne Research Inc.) analyzer was used for measuring nitrous oxide (in ppb).

### Measurement protocol and soil analysis

Gas measurements were done five days each week for the first two weeks from the day of soil microcosm preparation. Then two days per week in the two successive weeks and one day per week in the last three-four weeks.

Before and after each gas measurement, masses of the soil microcosms were recorded. Water loss from microcosms during measurements were compensated by adding equivalent amounts of ultra-pure water to the incubation cylinders. Since the water loss after each measurement cycle was minimal, water was added weekly during the first two weeks and then once every two weeks until the end of incubation period.

The sampling of mineral N was done in the complementary cylinders prepared specifically for mineral N measurements at days 4, 7, 14, 28 and 50. Soil of each cylinder was mixed and packed in a labelled sachet and frozen under −20 °C until extraction. For extraction, the frozen samples thawed overnight at 5 °C. Airtight bags were prepared and 50 g of soil from each sample was added with 100 ml of 1 mol l^−1^ KCl (Potassium Chloride, 99% pure, Merck Chemicals) solution. They were then mixed in a rotative shaker for 30 min. The bags were then left standing undisturbed for 30 min for sedimentation. Vacuumed tubes were labelled and then used for collecting the supernatant with a syringe. The tubes were then frozen and later analyzed by colorimetric method for mineral NH_4_^+^ and NO_3_^-^.

### Calculation and statistical analysis

Cumulated daily fluxes were calculated until day 50 of incubation period to yield gaseous fluxes. Linear interpolation was used to fill the gaps between the 15 measurement days, by taking the mean of the daily gaseous flux between the precedent and the successive measurement day for each modality and their repetitions. The means were calculated from the three repetitions of each modality. We statistically compared the cumulative gaseous fluxes obtained between different vertical distributions and within each soil by a Tukey’s Honestly Significant Difference (HSD) post-hoc test. This test is a multiple comparison procedure used after a significant result from an Analysis of Variance (ANOVA) to control the family-wise error rate. The test compares all possible pairs of means and is based on the studentized range distribution (q). To account for multiple comparisons, the p-values from this test are adjusted. A difference between two groups is considered statistically significant if its adjusted p-value is below the predetermined alpha level (p < 0.05).

Principal Component Analysis (PCA) was performed to determine interrelationship between different gaseous fluxes and soil mineral N processes. The numerical variables selected for the analysis were the emissions and N pool measurements (NH_4_^+^ and NO_3_^-^ concentrations in the soil, CO_2_, N_2_O and NH_3_ fluxes). The selection only included the days where soil mineral nitrogen concentrations were measured (4, 7, 14, 28 and 50). Categorical variables (soil type, residue modality, replicate and Day) were retained for grouping and statistical modeling. The total dataset comprised a total of n = 180 observations per variable. The selected numerical variables were standardized (scaled to have a mean of 0 and a standard deviation of 1) before PCA. PCA was conducted in R (version 4.5.0 (2025–04–11)) using the prcomp function from the stats package, and visualized using functions from the factoextra package. The resulting principal components (PCs) were interpreted based on their eigenvalues (proportion of variance explained) and the loadings of the original variables onto each PC. The first two principal components (PC1 and PC2) were chosen for detailed interpretation and subsequent statistical analysis as they collectively explained the majority of the total variance. To statistically evaluate the effects of residue modality and soil type on the identified principal components, a two-way Analysis of Variance (ANOVA) was performed. The score of the first principal component (PC1), was used as the dependent variable in the ANOVA. Residue modality (categorical, with levels: control, mixed, surface and sandwich) and Soil (categorical, with levels: GICOS, GEXER and SLU) were treated as independent fixed factors. The ANOVA model was specified as PC1 ~ Residue * Soil, including main effects for Residue and Soil, and their interaction. Tukey’s Honestly Significant Difference (HSD) post-hoc tests were conducted to perform pairwise comparisons between group means.

## Results

Overall, N_2_O and NH_3_ emissions exhibited similar temporal patterns but were differently influenced by soil type and residue placement. SLU soil had the highest N_2_O and NH_3_ emissions. N_2_O emissions were the most significant in the mixed and layered residue placement whereas NH_3_ emissions only occurred in the surface treatment.

### Temporal dynamics of N_2_O emissions

#### Cumulative emissions

**Fig. 2:**
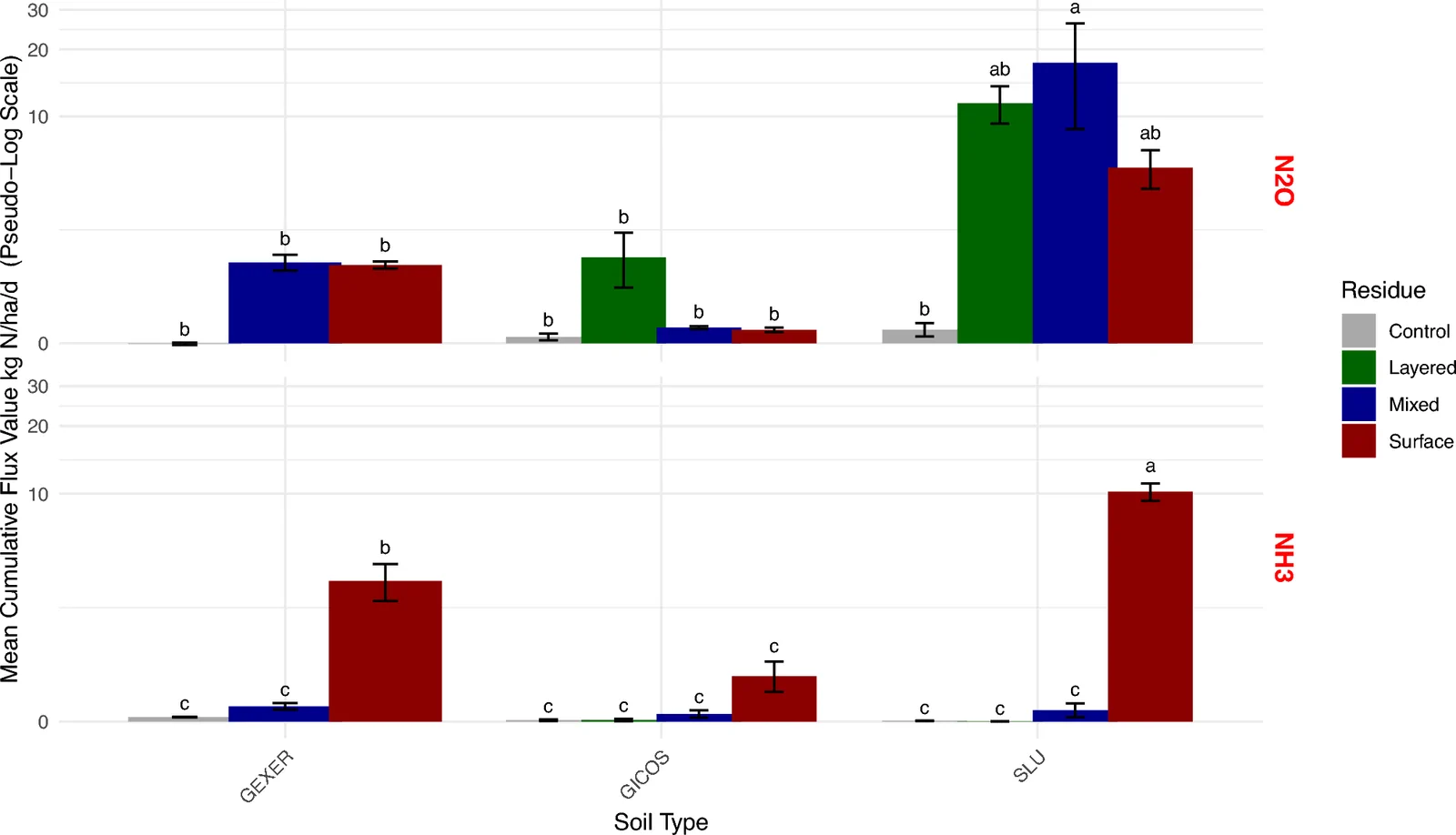
Mean and standard deviation of cumulative N_2_O (top) & NH_3_ (bottom) fluxes at day 50 by soil type and residue treatment. SLU: Swedish soil, GEXER: Grignon Exercise French Soil, GICOS: Grignon ICOS site French Soil. Small letters represent results of the Tukey HSD Post-hoc Test on combined treatments. Y axis is in pseudo log scale.

Swedish soil SLU had the highest N_2_O cumulated flux for 50 days of incubation (Fig. 2). Within SLU modalities, red clover mixed on the top half of soil had highest average cumulated N_2_O flux of 17.4 kg N ha^−1^. Next followed layered modality with net average cumulated N_2_O flux of 11.5 kg N ha^−1^ and red clover on surface had the lowest average net cumulated N_2_O flux of 5.8 kg N ha^−1^. In the case of GICOS soil, layered modality recorded the highest average cumulated N_2_O flux of 2.0 kg N ha^−1^, mixed and surface modality subsequently recorded average net cumulative flux of 0.3 kg N ha^−1^ and 0.3 kg N ha^−1^_._ For GEXER soil both surface and mixed microcosms had statistically similar average net cumulated N_2_O fluxes of 1.9 kg N ha^−1^and 1.8 kg N ha^−1^.

#### Temporal profiles

**Fig. 3:**
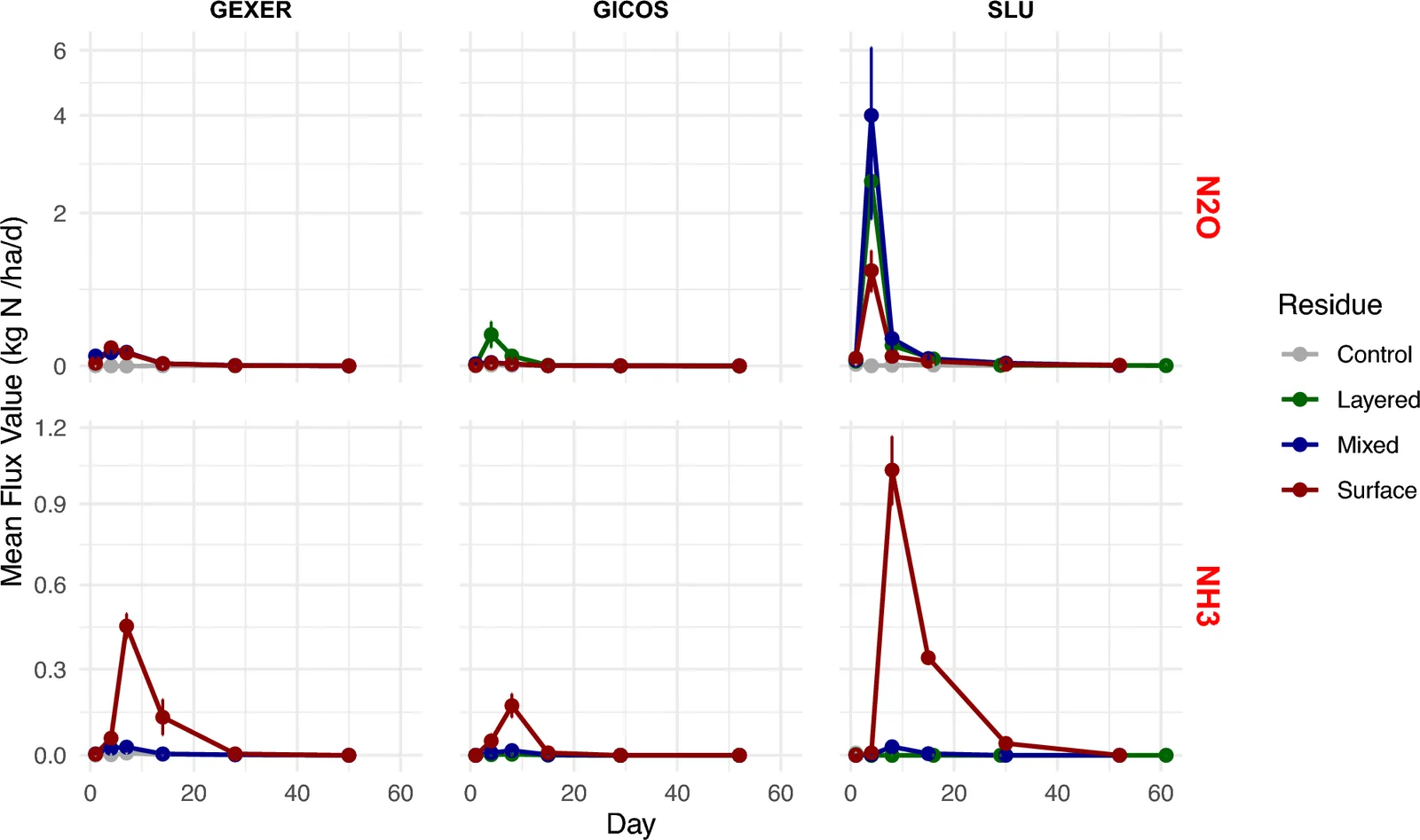
Dynamics of mean and standard deviation of N_2_O and NH_3_ fluxes for surface, mixed, layered and control modalities for the three soil types (SLU, GEXER and GICOS). SLU: Swedish soil, GEXER: Grignon exercise French soil, GICOS: Grignon ICOS site French soil. Y axis is in pseudo log scale.

N_2_O fluxes reached their peak values at day 4 for all soils and modalities except for GEXER mixed modality which was reached on day 7 (Fig. 3). The highest peak value of daily flux was recorded for SLU soil at 4.0 kg N ha^−1^ d^−1^ for the mixed modality. GICOS soil had the intermediate peak daily N_2_O flux at 0.4 kg N ha^−1^ d^−1^ for the layered modality, while GEXER soil had the lowest peak at 0.2 kg N ha^−1^ d^−1^ for the surface modality. From day 7 to the end of incubation, the measures N_2_O fluxes for three soils and residue modality phase towards zero.

#### Influence of placement and soil type

French soils GICOS and GEXER had similar N_2_O cumulated fluxes, which did not differ statistically according to the Tukey test (Fig. 2). Whereas SLU fluxes were different and the mixed modality was significantly different than the other modalities.

### Temporal dynamics of NH_3_ emissions

#### Cumulative emissions

Significant fluxes of NH_3_ were measured for the three soils concerning the surface modality (Fig. 2). Surface modalities had the highest emissions with mean cumulative flux values of 10.2 kg N ha^−1^, 3.9 kg N ha^−1^ and 1.0 kg N ha^−1^ respectively for SLU, GEXER and GICOS soils. Mixed and layered modalities recorded near-zero net cumulated NH_3_ fluxes.

#### Temporal profiles

NH_3_ flux dynamics reached peak values at day 7 for GEXER soil and day 8 for SLU and GICOS soils for the surface modality. SLU soil recorded the highest peak daily NH_3_ flux at 1.0 kg N ha^−1^ d^−1^, followed by GEXER soil at 0.5 kg N ha^−1^ d^−1^ (Fig. 3). GICOS soil had the lowest NH_3_ daily flux peak, at 0.2 kg N ha^−1^ d^−1^.

### Carbon dioxide fluxes

**Fig. 4:**
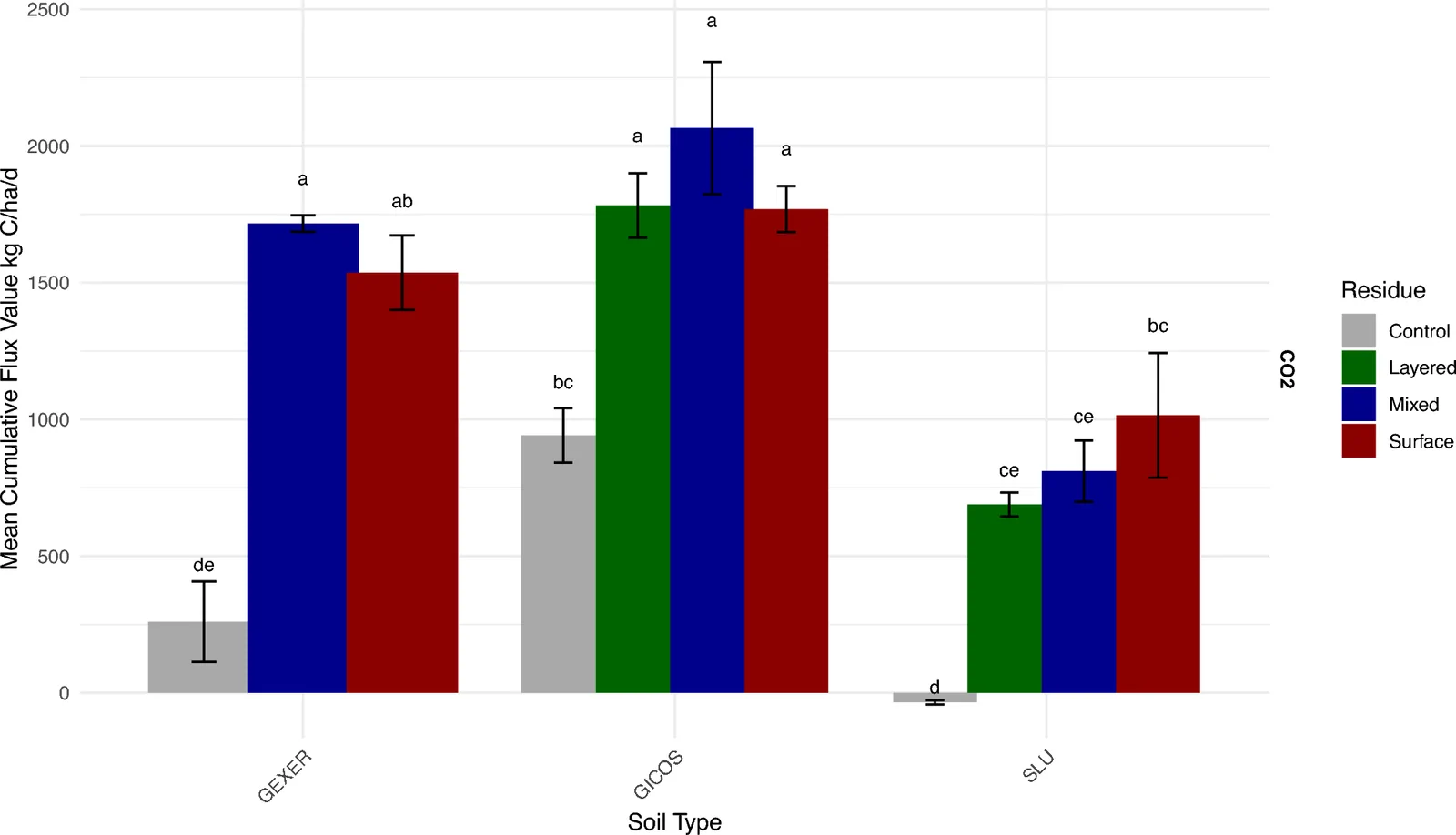
Mean and standard deviation of cumulative CO_2_ fluxes at day 50 by soil type and residue treatment. SLU: Swedish soil, GEXER: Grignon Exercise French Soil, GICOS: Grignon ICOS site French Soil. Small letters represent results of the Tukey HSD Post-hoc Test on combined treatments.

The cumulative emissions for CO_2_ are reported in Fig. 4. We do not note a statistical difference between different residue placement positions for all three soils. However cumulated fluxes were statistically similar for GICOS and GEXER with residue addition and significantly higher than SLU soil. Control soils with no residue addition had significantly lower CO_2_ cumulated fluxes for all three soil types.

**Fig. 5:**
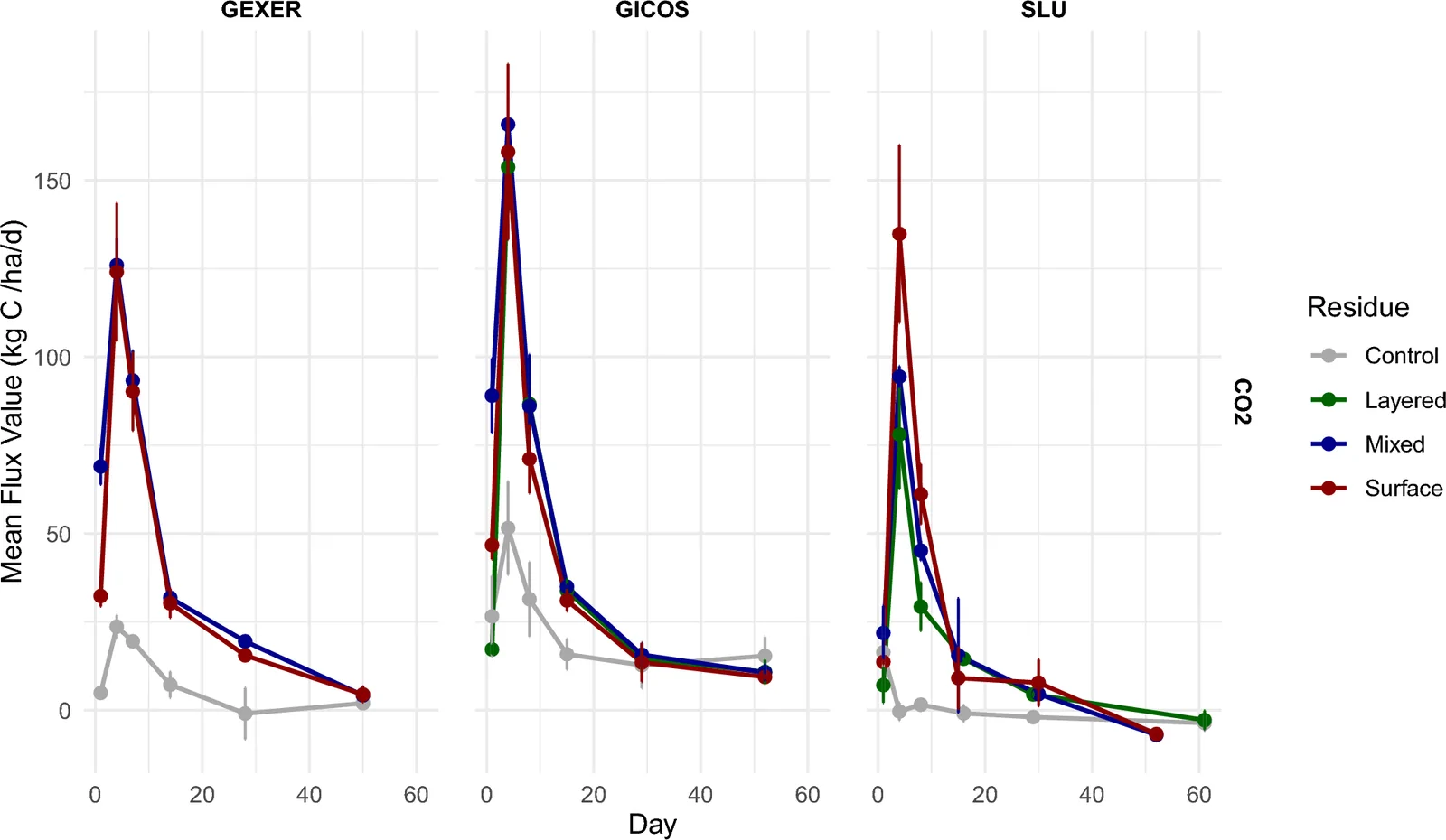
Dynamics of mean and standard deviation of CO_2_ fluxes for surface, mixed, layered and control modalities for the three soil types (SLU, GEXER and GICOS). SLU: Swedish soil, GEXER: Grignon exercise French soil, GICOS: Grignon ICOS site French soil.

Concerning CO_2_ daily fluxes, the maximums were registered for GICOS soil at 165. 8, 158.0 and 153.7 kg C ha^−1^ d^−1^ on day 4 for the mixed, surface and layered modalities respectively. SLU soil had a peak at 134.4 kg C ha^−1^ d^−1^ for the surface modality followed by 94.4 kg C ha^−1^ d^−1^ for the mixed modality and 78.0 kg C ha^−1^ d^−1^ for the layered modality all occurring on day4 (Fig. 5). GEXER soil had CO_2_ flux at 126.0 kg C ha^−1^ d^−1^ on day 4 for the mixed modality and 124.0 kg C ha^−1^ d^−1^ on day 4 for the surface modality.

Mixed modalities had higher peaks than the surface modalities for calcareous and silt-loamy GICOS and GEXER soils. However, surface modality has higher peak than mixed modality in sandy SLU soil. The control modality of GEXER soil reached a slight peak at 23.6 kg C ha^−1^ d^−1^ on day 4 and GICOS control modality at 60.0 kg C ha^−1^ d^−1^ on day 3. SLU control CO_2_ daily flux remained close to zero.

### Soil mineral N content changes

**Fig. 6:**
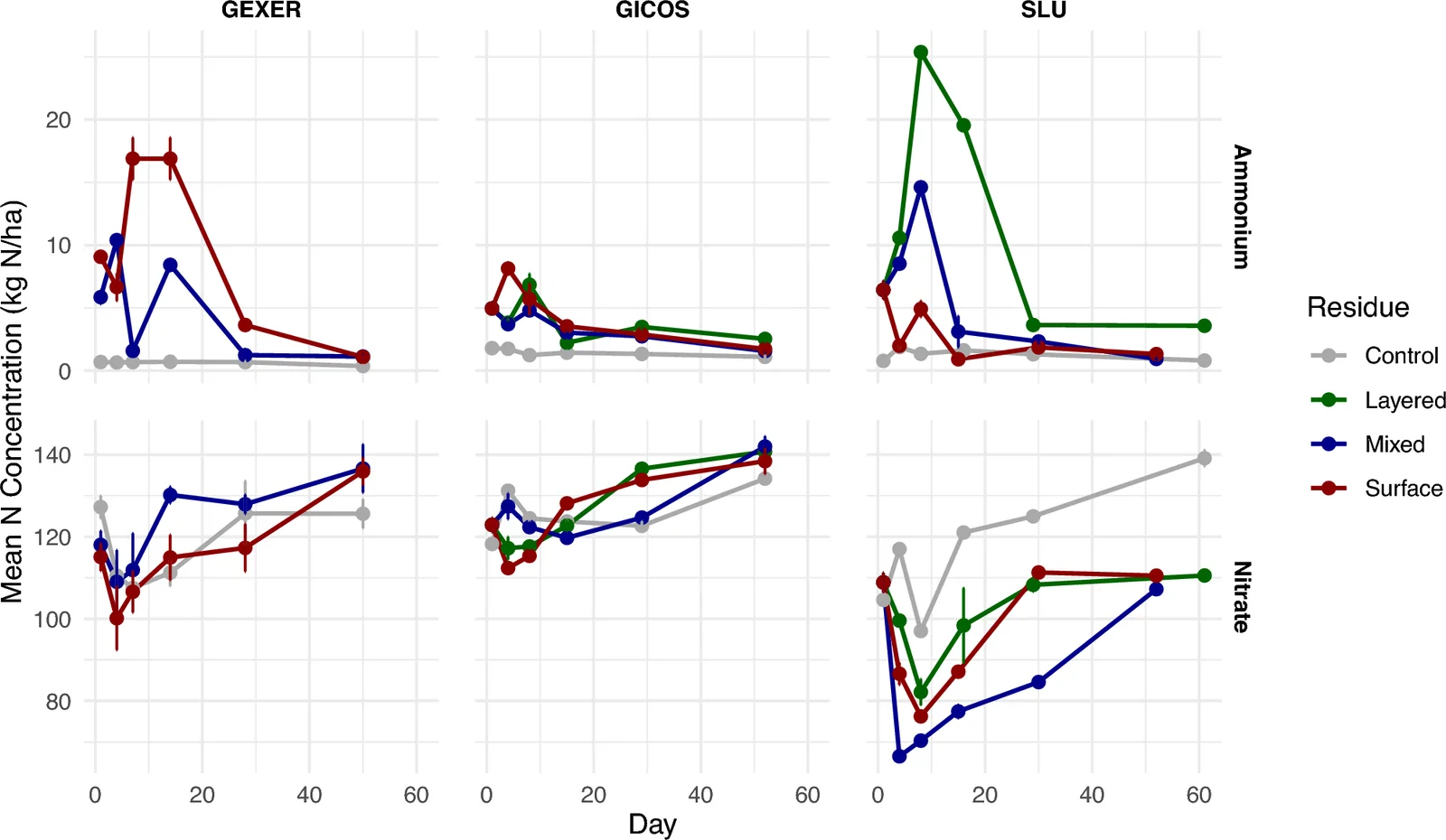
Mean and Standard deviations of ammonium (NH_4_^+^) and nitrate (NO_3_^-^) concentrations in kg N ha-1 measured in soil cores at different dates and for different soil types and residue modalities. SLU: Swedish soil, GEXER: Grignon Exercise French Soil, GICOS: Grignon ICOS site French Soil.

The application of different residue treatments significantly influenced soil NH_4_^+^ and NO_3_^−^ concentrations across three distinct soil types over a 50-day period (Fig. 6).

In the GEXER soil, NH_4_^+^ concentrations in the mixed and surface treatments displayed a rapid and substantial increase, peaking at 16.5 kg N ha^−1^ at day 14 and 10.1 kg N ha^−1^ at day 4 for surface and mixed treatments respectively, before declining to 0.4 and 1.1 kg N ha^−1^ by the end of the experiment. All residue treatments resulted in a decrease in NO_3_^-^ concentrations until day 7 followed by and an increase over time until day 50.

The GICOS soil responded differently, with residue treatments causing a more moderate increase in NH_4_^+^. The mixed and layered treatments both peaked on day 7 at 4.7 and 6.7 kg N ha^−1^, respectively, while the surface treatment reached its maximum concentration of 7.9 kg N ha^−1^ on day 4. Nitrate concentrations in this soil type and for the surface and layered treatments showed a similar trend to GEXER soil with a decrease until day 4 followed by an increase with concentrations reaching 135.0 and 137.2 kg N ha^−1^ respectively. A slight increase in NO_3_^-^ concentrations for the mixed treatment was observed although it was not statistically different before it reaches its minimum on day 14 and then increasing again to reach values similar to both other treatments.

In the SLU soil, the impact of residue treatments on N dynamics was distinct from the other soil types. The mixed and layered treatments caused a peak in NH_4_^+^ on day 7 reaching values of 14.2 and 24.7 kg N ha^−1^ respectively. In contrast, the surface treatment did not show much change in NH_4_^+^ content. All residue treatments in the SLU soil resulted in a decrease in NO_3_^-^ concentrations until day 7 followed by an increase though not exceeding initial values.

Across all soil types, the control groups consistently maintained the lowest concentrations of NH_4_^+^ often very close to zero. As for NO_3_^-^, the concentrations followed the same trend as for the residue’s treatments with slightly higher concentrations in the SLU soil.

### Relationships between soil mineral N content, N_2_O, CO_2_ and NH_3_ fluxes

To explore the relationships between the different gas fluxes in relation to soil mineral N content, as well as soil properties and vertical residue, distribution we conducted a PCA. The PCA was used as an exploratory tool to identify co-variation among the multiple response variables measured simultaneously. The eigenvalues and explained variance ratios are presented in Table 3.

**Table 3:** Explained variance by principal components.

PC	Eigen value	Explained variance ratio (%)	Cumulative explained variance (%)
PC1	1.937	38.75	38.75
PC2	1.037	20.73	59.48
PC3	0.824	16.47	75.95
PC4	0.757	15.14	91.09
PC5	0.445	8.91	100.00

The first principal component (PC1) explained 38.7% of the total variance, while the second principal component (PC2) explained an additional 20.7%. Together, PC1 and PC2 accounted for 59.48% of the total variance, suggesting that these two components capture the majority of the variability in the emission and N pool data. The eigen values of the original variables on the principal components indicate the contribution and direction of each variable to the respective PC as shown in Table 4. We note that PC1 is primarily characterized by strong positive loadings from CO_2_, N_2_O and NH_4_^+^. This suggests that PC1 represents an "overall biological activity" or “decomposition intensity” axis, where higher values indicate increased microbial activity and associated gas emissions and N transformations. Conversely, NO_3_^-^ might have a negative loading, indicating an inverse relationship with this axis. PC2, on the other hand, shows strong positive loadings from NH_3_ and negative loadings from NO_3_^-^, CO_2_ and N_2_O. This indicates that PC2 represents the dominance of NH_3_ volatilization versus NO_3_^-^ accumulation or denitrification or the prevalence of a more physico-chemical effect related to NH_3_ volatilization with a large NH_3_ component and a low NH_4_^+^ component.

**Table 4:** Variable loadings (Eigenvectors) on principal components.

Variable	PC1	PC2	PC3	PC4	PC5
*NH*_*4*_^+^	0.4830	0.0470	−0.2412	0.7670	−0.3436
*NO*_*3*_^*-*^	−0.5556	−0.1389	−0.4606	−0.0878	−0.6724
CO_2_	0.4219	−0.2470	−0.7463	−0.3681	0.2617
N_2_O	0.4203	−0.5753	0.4155	−0.3105	−0.4726
NH_3_	0.3215	0.7658	0.0027	−0.4149	−0.3714

**Fig. 7:**
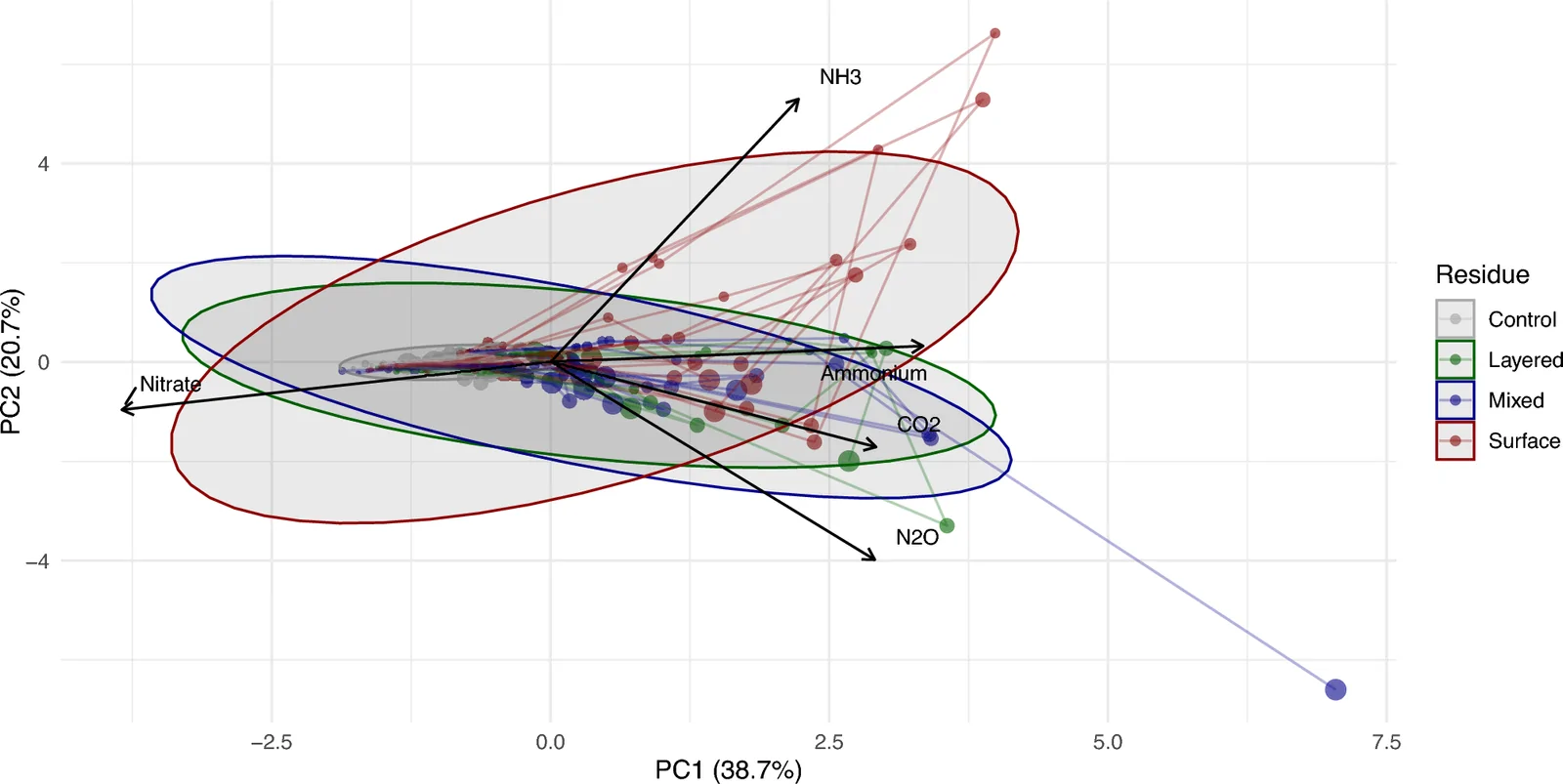
PCA scores with temporal trends by residue treatments with variable arrows projected onto the first two principal components PC1 and PC2. The arrows originating from the center of the plot represent the variable loadings (or correlations). The individual points are the scores, representing the position of each specific measurement (Soil Type x Residue x Day x Replicate) within the principal component space. The points are colored by the Residue Treatment. The size of the points is scaled by Day (larger points for Day 1). The thin lines connecting the points track the trajectory of a single replicate’s profile over the duration of the experiment.

The biplots (Fig. 7) visually represent the relationships between residue treatment and variables in the PC1-PC2 plane, including the correlation circle. Samples tend to cluster based on residue treatment. For instance, mixed residue treatment appears to be associated with higher CO_2_ and N_2_O values (pointing in the same direction as the PC1 axis), while surface residue treatment is associated with NH_3_ fluxes. We did not distinguish clustering for soil types.

Figure 7 also shows the temporal trends. The plots showing PC1 vs. PC2 with points sized by Day and connected by lines for each replicate reveal temporal trajectories. We note that samples generally move from lower PC1 values at early days to higher PC1 values around Day 7–15, indicating an initial surge in biological activity, followed by a decline. These visual trends suggest that both residue treatment and soil type influence the temporal evolution of emission patterns.

### Effects of residue, soil and mixed residue:soil effects on overall emissions

The results (Table 5) indicate that there was a statistically significant main effect of residue on PC1. This suggests that different residue treatments significantly influence the overall biological activity and emission patterns. There was also a statistically significant main effect of soil on PC1. This implies that the inherent properties of the soil types significantly affect the overall biological activity. The interaction between residue and soil was less clear. A significant interaction means that the effect of residue treatment on overall biological activity depends on the soil type, and vice-versa. This highlights the importance of considering both factors together and the use of PCA.

**Table 5:** ANOVA Summary for PC1 (overall biological activity).

Source of Variation	Degree of freedom	Sum of squares	Mean Square	F value	p value	Significance
Residue	3	58.2	19.399	13.885	3.35E-08	***
Soil	2	41.26	20.631	14.768	1.13E-06	***
Residue:Soil	5	19.32	3.865	2.766	0.0196	*
Residuals	184	257.06	1.397			

*** p < 0.001, ** p < 0.01, * p < *0.05,. p* < *0.1*.

From Table 6, it is evident that control with no residues resulted in significantly different overall biological activity compared to the three modalities of residues. However, there was no significant difference between surface, layered and mixed residue treatments. This suggests that residue incorporation methods generally alter the overall biological activity compared to no residue, but their effects on PC1 are not statistically distinct from each other. This would mean the “biological activity” is not dependent on the way it is incorporated confirming what was observed with the soil respiration rates where fluxes were similar for different residue modalities.

**Table 6:** Tukey HSD for Residue on PC1 (Adjusted p-values).

Contrast	p adj	Significance
Layered-Control	0.000018	***
Mixed-Control	0.000006	***
Surface-Control	0.000001	***
Mixed-Layered	0.992908	
Surface-Layered	0.997175	
Surface-Mixed	0.953404	

*** p < 0.001, ** p < 0.01, * p < *0.05,. p* < *0.1*.

Table 7 indicates a highly significant difference in overall biological activity (PC1) when comparing with SLU soil. However, GEXER and GICOS soils did not show any significant differences between them from a biological activity point of view (p = 0.06). Specifically, SLU soil showed higher PC1 scores compared to GICOS or GEXER soil, suggesting fundamental differences in their microbial activity and emission potentials. There is a high degree of similarity observed among the Surface and Mixed conditions, particularly for the SLU and GICOS soils. On the other hand, the near-zero similarity of the Control modality in all soils validates its effectiveness as a distinct and non-correlated baseline. The slight similarity between the Control:GICOS and Control:GEXER data points suggests that those soils might have similar characteristics.

**Table 7:** Tukey HSD for Soil on PC1 (Adjusted p-values).

Contrast	p adj	Significance
GICOS-GEXER	0.0644304	
SLU-GEXER	0.0157617	*
SLU-GICOS	0.0000005	***

*** p < 0.001, ** p < 0.01, * p < *0.05,. p* < *0.1*.

## Discussion

The strategic management of crop residues is a central issue in sustainable agriculture, balancing the benefits of soil health enhancement against the potential for increased gaseous N losses. This study aimed to quantify the temporal dynamics of N_2_O and NH_3_ emissions from a N-rich residue (red clover) under three different vertical distribution patterns (mixed, layered, and surface) across three distinct soil types. Our findings largely support the initial hypothesis that both the magnitude and temporal patterns of N_2_O and NH_3_ emissions are significantly influenced by the interaction between residue placement and soil properties.

###  N_2_O emissions and the role of residue placement within soil

The study data show a clear and significant increase in cumulative N_2_O emissions when red clover residues were incorporated into the soil (mixed and layered modalities) compared to being left on the surface as mulch. This is particularly evident in the SLU soil, where the mixed modality resulted in a significantly higher cumulative N_2_O flux than the surface treatment (17.4 kg N ha^−1^ vs. 5.8 kg N ha^−1^). This finding is consistent with previous research demonstrating that incorporating organic matter into the soil creates localized “hot spots” for microbial activity due to enhanced microbial contact and decomposition (^26^,^53,54^). The intimate contact between the labile red clover and the soil provides optimal conditions for a rapid surge in microbial decomposition, which fuels nitrification and denitrification processes that produce N_2_O^55–57^. The temporal dynamics of N_2_O emissions, with peak fluxes occurring within the first week of incubation for almost all treatments, align with this concept of rapid N cycling^58–60^. The subsequent decline in fluxes after day 7 suggests that the most readily available C and N from the residue have been processed, and microbial activity has returned to a more stable state. The concurrent peak in daily CO_2_ flux further supports this idea of intense heterotrophic decomposition. This interpretation is also supported by the PCA, which identified a primary axis (PC1) representing overall biological activity, characterized by strong positive loadings from CO_2_, N_2_O, and soil NH_4_^+^ concentrations.

The pronounced differences in N_2_O fluxes among the three soil types, with SLU soil showing significantly higher emissions than the French soils (GEXER and GICOS), underscore the critical role of soil properties related to physico-chemical composition but also to the microbial communities present. Our results align with other laboratory experiments^60–62^that suggest higher emissions from coarse-textured sandy soils compared to fine-textured silty soils. Taghizadeh-Toosi et al.^51^ also reported similar N_2_O emissions from the same SLU soil with Red Clover addition in two different placements. While Lashermes et al.^41^ found higher emissions from the SLU soil with mixed red clover compared to GICOS soil confirming our results. This might be explained by the higher gaseous diffusivity in the sandy SLU soil allowing for greater oxygen availability, promoting decomposition and releasing more NH_4_^+^ ions for nitrification and aerobic denitrification. Fine-textured soils (GEXER and GICOS), with their low diffusivity, would be more oxygen-deficient, facilitating anaerobic denitrification and the complete reduction to N_2_, thus leading to lower N_2_O emissions. Additionally, the slightly acidic pH of the SLU soil (6.2) favors the stabilization of NH_4_^+^ but also limiting the reduction of N_2_O to N_2_ a factor that has been demonstrated to lead to higher N_2_O flux^63^,^64^. This contrasts with some field-level studies^65,66^ that typically observe higher N_2_O fluxes in fine-textured soils, a discrepancy often attributed to differences in water retention in field conditions versus our controlled laboratory setting with constant 60% WFPS. The significant main effect of “Soil” on the overall biological activity (PC1) confirmed by the ANOVA further highlights that soil properties are a primary determinant of emission patterns.

###  NH_3_ emissions: a trade-off with residue placement

In contrast to the N_2_O findings, significant NH_3_ volatilization was almost exclusively limited to the surface-placement modality. The mixed and layered treatments recorded near-zero net cumulative NH_3_ fluxes, which contradicts the second part of our hypothesis stating that incorporated residues (mixed and layered) could initially lead to high NH_3_ volatilization due to enhanced microbial contact and decomposition. This is strongly supported by previous studies showing that residue incorporation is an effective strategy for significantly reducing, or even eliminating, NH_3_ losses^29^. When residues are placed on the soil surface, the N released during decomposition is exposed to the atmosphere, where it can be lost as NH_3_ gas. In the mixed and layered modalities, the NH_3_ released is likely trapped within the soil layers^67^. Our PCA results clearly separate NH_3_ emissions from other biological activity, with NH_3_ having a strong positive loading on the second principal component (PC2) while other gases like N_2_O and CO_2_ have negative loadings. This confirms that NH_3_ volatilization is driven by different mechanisms than the microbial decomposition pathways that produce N_2_O since it is an equilibrium process controlled by physical and chemical drivers (boundary layer resistance and the acido-basic and Henry equilibria).

The temporal dynamics of NH_3_ emissions also differed from N_2_O, with peak values obtained in the second week of incubation for all surface modalities, a delay of around 4–5 days from the peak of N_2_O flux. Other laboratory experiments have also observed this delay^61,67^, which is likely related to the rise in soil NH_4_^+^ content in the first week.

Regarding the role of soil type, our results are consistent with the proposition that soils with high clay content result in lower NH_3_ emissions due to ionic adsorption^68,69^. The sandy SLU soil, with its higher proportion of coarse particles, had the highest net cumulative NH_3_ flux, while the excessively silty GICOS soil had the lowest. The observation that the slightly acidic SLU soil (pH 6.18) exhibited the highest NH_3_ emissions is less obvious since the general understanding that this physiochemical process is favored by high soil pH which shifts the chemical equilibrium from the non-volatile NH_4_^+^ ion to the volatile NH_3_ gas, which is subsequently volatilized^70^. This may be explained by the sandy texture of the SLU soil, which could promote faster gas diffusion from the surface residues, by the possible different pH values on the soil surface and within the crop residues, or by the possible lower nitrification rate leading to NH_4_^+^ accumulation. The lower nitrification rate observed in the SLU sandy loam soil, despite its presumably more aerobic conditions, can be explained by several interacting factors. (1) Soil pH: SLU has a markedly lower pH (6.2) compared to GEXER (8.3) and GICOS (7.6). Nitrifying bacteria, particularly ammonia-oxidizing bacteria (AOB), generally exhibit lower activity at acidic pH values. (2) Faster N₂O emissions: The SLU soil showed by far the highest N₂O emissions, indicating that a substantial fraction of the mineral N was lost via denitrification or nitrifier denitrification rather than accumulating as NO₃⁻. (3) NH₄⁺ availability: In the SLU surface treatment, significant NH₃ volatilization also removed N from the system.

###  CO_2_ fluxes, residue placement, and mineralization dynamics

The CO_2_ fluxes provided a measure of the overall heterotrophic microbial decomposition intensity. The rapid and high peak fluxes observed around Day 4 confirm a “hot moment” of activity driven by the rapid consumption of the labile C fraction in the red clover residue (Fig. 5). The addition of 100 mg kg^−1^ NO_3_^-^ for the pre-incubation of soils ensured that microbial decomposition was C driven, and not N limited. This is evidenced by the initial decrease in soil NO_3_^-^ concentrations (Fig. 6) as the microbial community rapidly immobilized mineral N to fuel the intense decomposition, especially in the SLU soil, where the control CO_2_ flux remained negligible. Across all soil types, the cumulative CO_2_ emissions were statistically similar regardless of residue placement (Fig. 4), suggesting that once the readily decomposable C is added, the overall magnitude of C respiration over 50 days is determined more by the quantity of the added substrate and the native soil C pool rather than whether the residue was mixed, layered, or on the surface. However, the significantly lower cumulative CO_2_ in the SLU soil compared to the French soils GEXER and GICOS suggests that the physicochemical soil characteristics (e.g., lower C content, Table 1) remain a key control on the baseline soil respiration, confirming that CO_2_ emissions are fundamentally rooted in the C saturation and microbial efficiency of the specific soil matrix. Comparing the net respired CO_2_ (treatement – control) of each treatement to the amount of fresh C added (1676 kg C ha^−1^) an average of 2.5, 1.7 and 1.5% of the added fresh C were respired as CO₂ over the 50-day incubation period under our experimental conditions, for the GEXER, GICOS and SLU soils respectively. The long-term fate of this carbon requires further investigation.

###  Residue emission factors for N_2_O

The calculated apparent Emission Factors (EF = cumulated fluxes for 50 days divided by the N input (100 kg N ha^−1^) provide a variable easily comparable to other studies. The EF for N_2_O values demonstrated extreme sensitivity to soil type and residue placement, with the highest EF reaching 17.4% in the SLU mixed treatment. This apparent EF represents an upper-bound, short-term laboratory EF under conditions optimised for N₂O production, not a value directly comparable to the IPCC EF for crop residue N. We note however that this is significantly higher than the IPCC default EF values of 0.6% (0.1–1.1%) and 0.5% (0.0–1.1%) for wet and dry climates^71^, respectively and provides a strong indictaion of the “hot moment” potential of red clover residues in sandy soils. This comparison is to consider carefully as IPCC EF are determined in long-term field conditions with fluctuating soil water content and temperatures. In the study of Janz et al^4^. who used the same red clover residue but a differrent soil under varied moisture, their EF values for incorporated residues (2.5%−6%) were generally lower than our maximum SLU mixed EF. This difference suggests that the specific combination of high WFPS (60%) and the non-limiting NO_3_^-^ pre-amendment in our experiment truly maximized the conditions for denitrification.

### Study limitations and implications for future research

This study was conducted under controlled laboratory conditions, which allowed for a detailed and precise quantification of emissions over time. However, this controlled environment also represents a limitation, as the results may not be directly transferable to the dynamic and complex conditions of a real agricultural field. Variations in temperature, precipitation, and aeration, which were held constant in this study, could significantly alter the magnitude and timing of emissions. Another limitation is the use of a single N-rich residue type (red clover). While this allowed for a focused analysis, it is important to acknowledge that residue from other crops, with different C:N ratios and lignin content, would likely produce different emission patterns. A broader investigation into the specific soil properties, beyond pH and texture, that contribute to the distinct emission patterns observed in the SLU soil could provide a more nuanced understanding of NH_3_ and N_2_O emissions from surface residues. Another limitation for the experiment is the absence of monitoring of total N in the soils which could have given a good indication of total immobilization and re-mineralization. Future research should focus on validating these findings in field-scale studies to account for the variability of natural conditions and to explore a wider range of crop residue types and their effects under different management practices.

## Conclusion

In conclusion, this research has provided a clear picture of the distinct impacts of green red clover residue placement within soil profile on N_2_O and NH_3_ emissions. We confirm the existence of a critical trade-off: residue incorporation, while highly effective at preventing NH_3_ volatilization, significantly increases the magnitude and intensity of N_2_O emissions in non-limiting conditions of N. Conversely, leaving residues on the soil surface, while reducing N_2_O losses, results in substantial NH_3_ volatilization. The study highlights the dominant influence of soil type, with the sandy SLU soil showing higher emissions, which contradicts some field-scale studies but is consistent with other laboratory findings. We provide also a valuable set of observations characterizing both N_2_O and NH_3_ emissions with a high temporal frequency therefore highlighting the dynamics of those emissions and quantifying potential “hot moments”. It is noteworthy that the experiment was conducted in conditions maximizing N_2_O and NH_3_ emissions. These results suggest that tailored residue management practices, which account for both residue placement and specific soil properties, are essential for minimizing N losses and achieving a more sustainable balance between soil health and environmental protection.

## Data Availability

The data supporting these results are available on the following link 10.57745/XRTI7P
